# A Case of Incomplete Heerfordt Syndrome Diagnosed Following Fever Onset

**DOI:** 10.7759/cureus.94234

**Published:** 2025-10-09

**Authors:** Motoyasu Nakamura, Keisuke Suzuki, Eriko Yoshida, Kenta Watanabe, Hiroto Sasage, Kenji Dohi, Satoshi Suzuki

**Affiliations:** 1 General Medicine Department, Tone Health Cooperative Society, Tone Central Hospital, Gunma, JPN; 2 Department of General Medicine, Kawasaki Health Cooperative Association, Kawasaki Kyodo Hospital, Kanagawa, JPN; 3 Department of Emergency, Disaster and Critical Care Medicine, Showa Medical University School of Medicine, Tokyo, JPN

**Keywords:** facial paralysis, fever, heerfordt syndrome, lymphadenopathy, sarcoidosis

## Abstract

Heerfordt syndrome is a rare clinical manifestation of sarcoidosis characterized by a combination of facial nerve palsy, parotid gland swelling, anterior uveitis, and fever. The classical (complete) form presents with all four features, whereas the incomplete form requires the presence of at least two of the following: anterior uveitis, parotid enlargement, or facial nerve palsy. Parotid gland involvement is often bilateral, and uveitis may cause ocular symptoms such as blurred vision. A 78-year-old female presented with fever, a one-year history of facial paralysis, and blurred vision that was managed symptomatically on an outpatient basis. Computed tomography conducted two weeks prior to admission revealed hilar and mediastinal lymphadenopathy, while laboratory tests revealed elevated angiotensin converting enzyme (ACE) level and sIL-2 receptors. Lymph node biopsy confirmed a non-caseating epithelioid cell granuloma, leading to a diagnosis of sarcoidosis. A subsequent ophthalmological evaluation revealed uveitis, establishing a diagnosis of Heerfordt syndrome (incomplete owing to the absence of parotid swelling). Her fever and fatigue improved without steroid therapy, and she was followed up as an outpatient. Sarcoidosis is characterized by multiple systemic symptoms, including respiratory, cardiac, skin, neurological, and ocular involvement. This case suggests that Heerfordt syndrome should be considered as a differential diagnosis in patients with blurred vision, facial palsy, or parotid swelling accompanied by fever, to ensure prompt systemic assessment to differentiate and treat sarcoidosis.

## Introduction

Sarcoidosis is a multi-organ disease of unknown cause characterized by the formation of granulomas in various organs. Intrathoracic lesions are observed in 90% of patients with sarcoidosis and are associated with symmetric bilateral portal lymphadenopathy and diffuse pulmonary nodules [1]. Nonspecific systemic symptoms of sarcoidosis include fever that, in most cases, is mild but can sometimes reach as high as 39-40 °C [2]. Neurological symptoms such as impaired consciousness are also common in patients with sarcoidosis, and these symptoms may be caused by various associated complications. Treatment for sarcoidosis has been reported to be effective for improving the accompanying neurological symptoms [3,4].

Heerfordt syndrome is a form of sarcoidosis characterized by a combination of facial nerve paralysis, parotid gland enlargement, and uveitis, accompanied by mild fever [5]. Cases with all three symptoms, which account for 0.3% of patients with sarcoidosis, are termed “complete Heerfordt syndrome." Cases with only two of the three characteristic symptoms are termed “incomplete Heerfordt syndrome” and account for 1.3% of sarcoidosis cases [6]. Herein, we report a rare case of incomplete Heerfordt syndrome.

This article was previously posted to the Authorea preprint server on March 26, 2025.

## Case presentation

A 78-year-old female with a medical history of hypertension, IgA nephropathy, facial nerve paralysis, blurred vision, and congestive heart failure presented to our hospital with the chief complaint of fever. Her medication regimen prior to admission included vonoprazan fumarate (20 mg), azilsartan (20 mg), amlodipine (5 mg), furosemide (20 mg), and trichloromethiazide (1 mg), all taken once daily after breakfast. She had no history of exposure to metals such as beryllium, nor had she been using anti-TNFα drugs or immune checkpoint inhibitors. Further, she experienced no family history of tuberculosis or local tuberculosis epidemic. Her only occupational history included working in lotus root processing.

One year prior to admission, the patient developed facial nerve paralysis and blurred vision and was followed up as an outpatient in the otolaryngology and ophthalmology departments. One year prior to admission, the patient developed facial paralysis and visual impairment and was examined by both an otolaryngologist and an ophthalmologist on an outpatient basis. She further experienced fatigue and neurological symptoms, including cognitive decline. Fourteen days prior to admission, she developed a fever of 38 °C, ultimately prompting her to visit our hospital.

On admission, her vital signs were as follows: Glasgow Coma Scale of E4V5M6, blood pressure of 120/69 mmHg, heart rate of 90 beats/min, respiratory rate of 18 breaths/min, body temperature of 37.9 ℃, and oxygen saturation of 100% on room air. Physical examination revealed no swelling, cervical lymph node tenderness, or pharyngeal redness. Heart sounds were regular, and no abnormal lung sounds were detected. The abdomen was flat and soft, without tenderness, and there was no lower limb edema.

Laboratory investigations revealed elevated levels of interleukin (IL)-2 receptor (3,670 U/mL) (IL-2 receptor: a blood marker reflecting immune system activation, often elevated in sarcoidosis) and angiotensin-converting enzyme (ACE, 27.4 U/L) (Table 1).

**Table 1:** Blood analysis results Abbreviations: Alb, albumin; ALT, alanine aminotransferase; Amy, amylase; APTT, activated partial thromboplastin time; AST, aspartate aminotransferase; BUN, blood urea nitrogen; CK, creatine phosphokinase; Cl, chloride; Cre, creatinine; CRP, C-reactive protein; γ-GTP, gamma-glutamyl transferase; Glu, glucose; Hb, hemoglobin; K, potassium; LDH, lactate dehydrogenase; Na, sodium; Plt, platelets; PT, prothrombin time; PT-INR, prothrombin time/international normalized ratio; RBC, red blood cell; T-bil, total bilirubin; TP, total protein; WBC, white blood cell; BNP, brain natriuretic peptide; TSH, thyroid stimulating hormone; Free-T3, free triiodothyronine; Free-T4, free thyroxine; IL-2 receptor, interleukin-2 receptor; ACE, angiotensin-converting enzyme; QFT, QuantiFERON-TB Gold test; SS-A antibody, anti-Sjögren's syndrome A antibody; SS-B antibody, anti-Sjögren's syndrome B antibody; ANA antibody, antinuclear antibody; MPO-ANCA, myeloperoxidase anti-neutrophil cytoplasmic antibody; PR3-ANCA, proteinase 3 anti-neutrophil cytoplasmic antibody; D-dimer, cross-linked fibrin degradation product

Item	Result	Reference value	
WBC	5200 /μL	3300–9000 /μL
RBC	372 x 10^4 /μL	380–520 x 10^4 /μL
Hb	11.2 g/dL	13.0–17.0 g/dL
Plt	21.6 x 10^4 /μL	13–36 x 10^4 /μL
TP	7.4 g/dL	6.5–8.0 g/dL
Alb	3.8 g/dL	4.0–5.0 g/dL
AST	16 IU/L	10–40 IU/L
ALT	10 IU/L	5–45 IU/L
γ-GTP	10 IU/L	10–45 IU/L
CK	108 IU/L	40–200 IU/L
T-bil	0.58 mg/dL	0.2–1.2 mg/dL
LDH	184 IU/L	120–230 IU/L
BUN	35.4 mg/dL	45–89 mg/dL
Cre	2.6 mg/dL	0.6–1.2 mg/dL
Na	138 mEq/L	135–145 mEq/L
K	4.2 mEq/L	3.5–5.0 mEq/L
Cl	102.4 mEq/L	98–108 mEq/L
Amy	118 IU/L	40–130 IU/L
CRP	0.14 mg/dL	<0.3 mg/dL
BNP	33.6 pg/mL	<18.4 pg/mL
Glu	94 mg/dL	70–109 mg/dL
TSH	0.77 μIU/mL	0.5–5.0 μIU/mL
Free-T3	1.89 pg/mL	2.3–4.0 pg/mL
Free-T3	1.37 pg/mL	1.0–1.7 pg/mL	
IL-2 receptor	3670 U/mL	123-572 U/mL
ACE	27.4 U/L	8.3–21.4 U/L	
Mycobacterium tuberculosis QFT	(–)	(–)
SS-A antibody	(–)	(–)
SS-B antibody	(–)	(–)
ANA antibody	(–)	(–)
MPO-ANCA	(–)	(–)
PR3-ANCA	(–)	(–)
PT	134.1%	70–130%
PTINR	0.86	0.85–1.15
APTT	25.9 s	25–38 s
D-dimer	2 μg/mL	<0.5 μg/mL
urine-Red blood cells	45721 /HPF	0–2 /HPF
urine-White blood cells	30–50 /HPF	0–5 /HPF
urine-Cylinders	+	–
urine-Bacteria	1+	–
Sputum culture	Oral commensal bacteria only	–
Sputum Grocott stain	Negative	Negative
Sputum India ink	Negative	Negative

**Figure 1:**
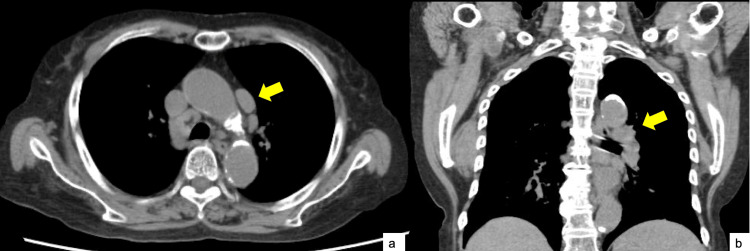
Results of plain chest computed tomography (CT) a: CT in the axial plane indicating mediastinal lymphadenopathy. b: CT in the coronal plane indicating hilar lymphadenopathy.

A plain chest computed tomography revealed swelling of the mediastinal lymph nodes (Figure 1).

**Figure 2:**
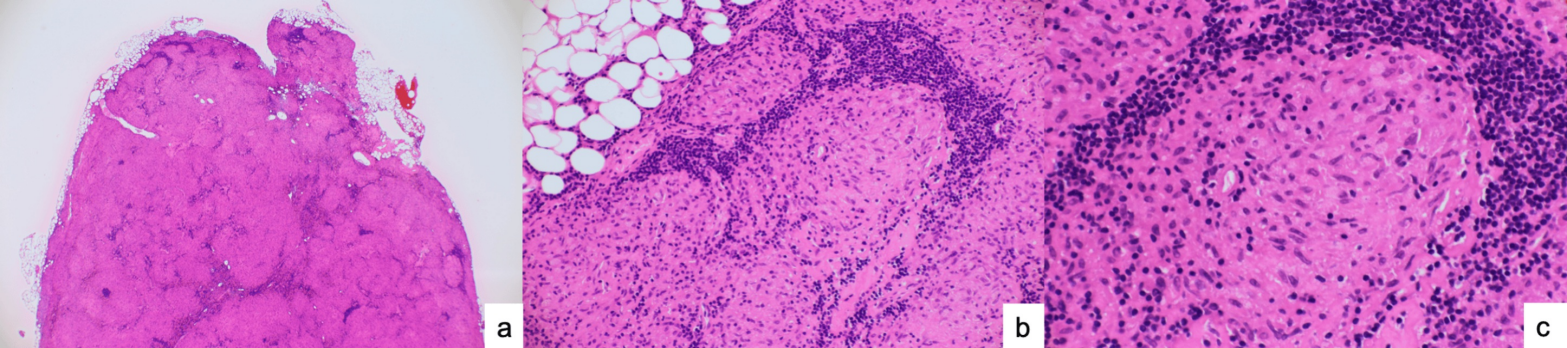
Histological analysis of biopsied lymph nodes a: Hematoxylin and eosin staining (2×). b: Hematoxylin and eosin staining (20×) indicating non-caseating epithelioid cell granulomas. No malignant tumor cells are identified. c: Hematoxylin and eosin staining (40×) indicating non-caseating epithelioid cell granulomas are observed. No malignant tumor cells are identified.

Pathological examination of the biopsied lymph nodes revealed non-caseating epithelioid cell granulomas and non-caseating granulomas (a type of granuloma without central necrosis, characteristic of sarcoidosis) (Figure 2).

**Figure 3:**
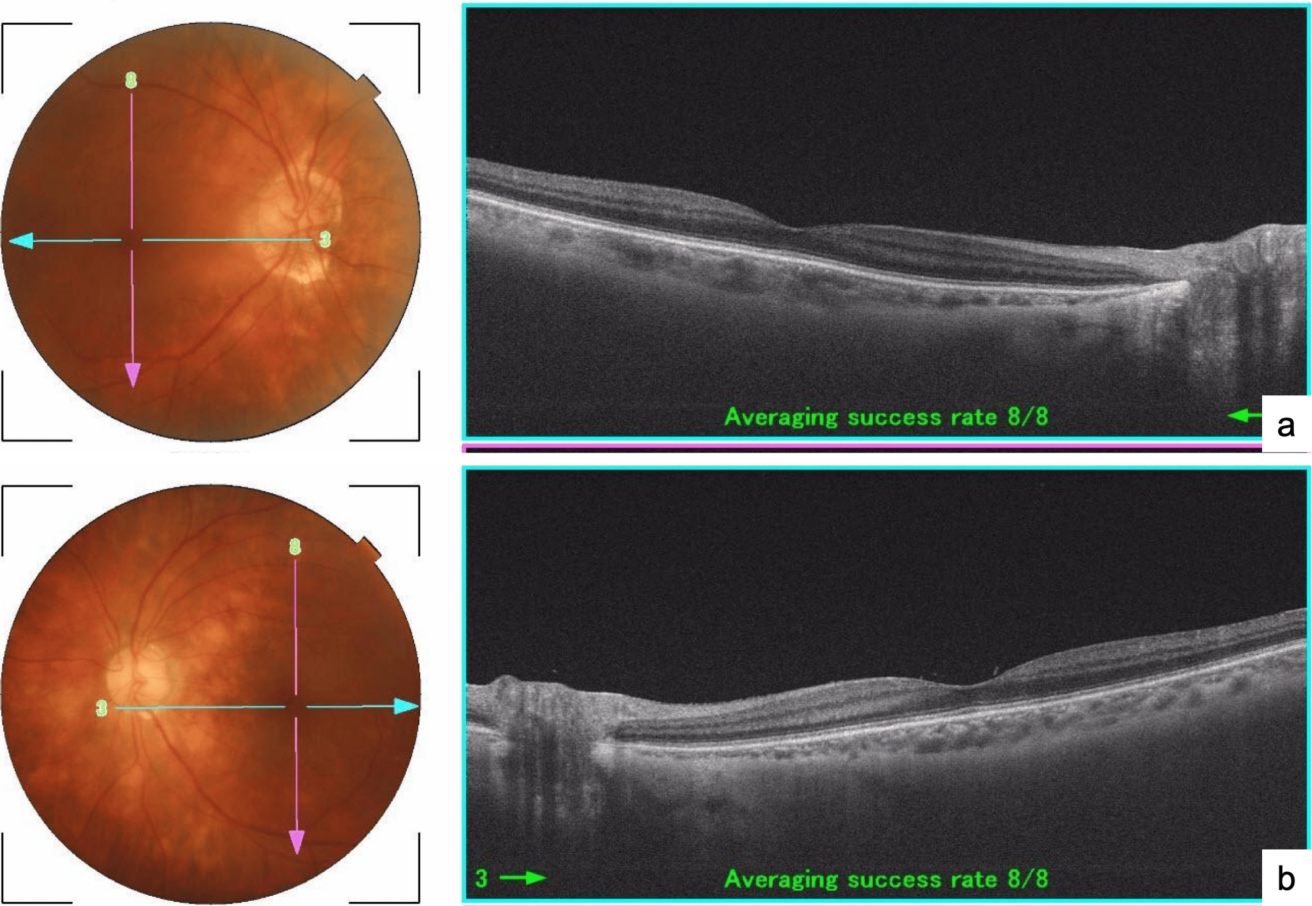
Results of ophthalmologic examination a,b: Retinal vasculitis and anterior chamber inflammation are evident as the faint opacity around the blood vessels along the optic nerve (a: right eye; b: left eye).

Ophthalmologic examination revealed a pale opacity around the blood vessels along the optic nerve, consistent with retinal vasculitis and anterior chamber inflammation, ultimately leading to a diagnosis of uveitis (Figure 3a, 3b).

The patient was initially treated with ceftriaxone (CTRX; 2 g/day) for pyuria and fever. However, the fever persisted despite antibiotic therapy, and subsequent imaging revealed progression of mediastinal lymphadenopathy. Based on the elevated IL-2 receptor and ACE levels in conjunction with the imaging findings, malignant lymphoma and sarcoidosis were considered in the differential diagnosis. Following the patient’s request for further evaluation, a surgical lymph node biopsy was performed on day 16 of hospitalization. This examination revealed a non-caseating epithelioid cell granuloma, confirming the diagnosis of sarcoidosis.

On day 23 of hospitalization, ophthalmological evaluation revealed membrane vasculitis with a faint perivascular opacity along the optic nerve and inflammation of the parotid gland, supporting the diagnosis of ocular sarcoidosis. Otorhinolaryngological examination performed on day 25 indicated no abnormal findings, while magnetic resonance imaging indicated no swelling of the parotid gland.

Although fever recurred intermittently during hospitalization, the patient exhibited signs of improvement, including following the natural course of fatigue, without the use of oral steroid therapy. Finally, the patient’s systemic symptoms improved, and she was discharged on the 42nd day of hospitalization to undergo outpatient follow-up without oral steroids. At discharge, the plan was to administer steroids if the patient’s symptoms worsened; however, her condition has remained stable to date (one year and three months post-discharge), with no further exacerbation of symptoms.

## Discussion

This case represents a rare occurrence in which a diagnosis of incomplete Heerfordt syndrome was ultimately made after a lymph node biopsy was performed on a mediastinal lymph node, despite a lack of accompanying parotid gland swelling.

A strict differential diagnosis is important for the diagnosis of sarcoidosis. Ensuring an accurate diagnosis further requires clarifying epidemiological factors such as local infectious diseases, travel areas, occupational and environmental exposure, drug abuse, exposure to medications, and family history [7].

In the present case, the pathological results revealed the presence of non-caseating granulomas. Although other diseases, including infectious diseases such as tuberculosis, autoimmune diseases, and malignant diseases, could be considered as differential diagnoses, sarcoidosis was ultimately diagnosed based on the test results.

Heerfordt syndrome was first described by Heerfordt and Waldenstrom in 1909, although its association with sarcoidosis was first reported only in 1937 [8,9]. As Heerfordt syndrome is a rare disease, no standard treatment strategy has yet been established. However, in cases with frequent facial nerve paralysis, corticosteroid treatment based on neurosarcoidosis is indicated [10]. Although the initial response rate to corticosteroids is high, symptoms may recur during corticosteroid tapering. In these cases, immunosuppressants such as azathioprine, methotrexate, cyclosporine A, and cyclophosphamide are applied in combination with corticosteroids [1]. In the present case, facial nerve paralysis persisted, and although steroid treatment was indicated, the patient refused to undergo steroid therapy. Therefore, we chose to observe the patient’s condition and to potentially administer steroid therapy based on their symptoms and preferences.

Sarcoidosis exhibits several clinical manifestations. Regardless of the presence or absence of symptoms, sarcoidosis can affect various organs, with clinical consequences ranging significantly from benign to very severe [1]. Previous reports have demonstrated that sarcoidosis is fatal in 5% of cases [11]. Incomplete Heerfordt syndrome is a rare disease; however, symptoms develop gradually and can sometimes appear as early symptoms of sarcoidosis [10].

 There are several limitations to this case report. First, as this is a single-case report, the generalizability of our observations remains limited, and our conclusions should be interpreted with caution. Second, the relatively short follow-up duration prevents us from drawing definitive conclusions regarding the long-term prognosis and potential recurrence of incomplete Heerfordt syndrome. Third, we were unable to perform advanced immunological or genetic analyses in this patient, and this limits our ability to explore detailed pathogenic mechanisms. Future studies should include systematic accumulation of similar cases, employ multicenter registries or databases, and incorporate comprehensive laboratory and imaging assessments to deepen our understanding of this rare presentation.

## Conclusions

Overall, in this report, we describe the diagnosis and management of a rare case of incomplete Heerfordt syndrome. Based on our experience with this case, we suggest that when fever is observed in addition to early symptoms such as facial nerve paralysis and parotid gland swelling, Heerfordt syndrome, a variant of sarcoidosis, should be considered. Subsequently, early systemic observation should be performed to differentiate these cases from sarcoidosis. However, as this report describes only a single case, caution should be exercised in generalizing these findings, and further studies are warranted to validate these observations.
